# On the Efficient Delivery and Storage of IoT Data in Edge–Fog–Cloud Environments

**DOI:** 10.3390/s22187016

**Published:** 2022-09-16

**Authors:** Alfredo Barron, Dante D. Sanchez-Gallegos, Diana Carrizales-Espinoza, J. L. Gonzalez-Compean, Miguel Morales-Sandoval

**Affiliations:** 1 Cinvestav Tamaulipas, Victoria 87130, Mexico; 2 ARCOS Research Group, Universidad Carlos III de Madrid, 28911 Leganes, Spain

**Keywords:** cloud storage, in-memory storage, edge–fog–cloud computing, data science

## Abstract

Cloud storage has become a keystone for organizations to manage large volumes of data produced by sensors at the edge as well as information produced by deep and machine learning applications. Nevertheless, the latency produced by geographic distributed systems deployed on any of the edge, the fog, or the cloud, leads to delays that are observed by end-users in the form of high response times. In this paper, we present an efficient scheme for the management and storage of Internet of Thing (IoT) data in edge–fog–cloud environments. In our proposal, entities called data containers are coupled, in a logical manner, with nano/microservices deployed on any of the edge, the fog, or the cloud. The data containers implement a hierarchical cache file system including storage levels such as in-memory, file system, and cloud services for transparently managing the input/output data operations produced by nano/microservices (e.g., a sensor hub collecting data from sensors at the edge or machine learning applications processing data at the edge). Data containers are interconnected through a secure and efficient content delivery network, which transparently and automatically performs the continuous delivery of data through the edge–fog–cloud. A prototype of our proposed scheme was implemented and evaluated in a case study based on the management of electrocardiogram sensor data. The obtained results reveal the suitability and efficiency of the proposed scheme.

## 1. Introduction

The amount of data produced in Internet of Things (IoT) environments has dramatically increased as IoT devices are constantly producing data [1,2]. The IoT data are hierarchically handled through the edge, the fog, the cloud, or any combination of these infrastructures [3]. In the edge [4,5], data are collected by using sensors to measure, for example, environmental data such as the weather [6,7] or health data (e.g., electrocardiogram signals [8]), whereas in the fog [9,10], the data are processed to obtain insights from the data-producing information by using data mining [11] and artificial intelligence [12] applications. Finally, in the cloud, data are stored and processed with big data and data science applications [13] to obtain further information useful in decision-making scenarios [14]. End-users access the information produced in the fog and the cloud by using visualization tools commonly developed as cloud services [15].

To perform the management and processing of IoT data [16,17,18], multiple applications are deployed on edge–fog–cloud infrastructures, which are organized in the form of processing structures (e.g., pipelines or workflows [19]). In these structures, the applications are managed by using directed acyclic graphs (DAG) [20], where nodes are the applications required for the processing/management of data (e.g., a QRS-complex detector when processing electrocardiogram data [21] or linear regressions when working with weather data for forecast [7]), whereas the edges represent the I/O dependencies between nodes.

In real scenarios of processing IoT data [16,17,18], the applications considered in the stages of processing structures, distributed in any of the edge, the fog, or the cloud, should be executed in an automatic manner to create a dataflow from the sensors to the cloud (and vice versa) [22,23] for supporting decision-making procedures [24].

In this sense, content delivery networks (CDNs) have been proposed to handle the delivery of data between applications distributed through multiple environments (any of the edge, the fog, or the cloud) [25,26]. Commonly, CDNs follow a centralized approach where the contents produced by a sensor or applications are stored in large storage servers (usually in the cloud or the fog) and then distributed to the applications/end-users that require the data for processing or visualization [27,28].

Nevertheless, when working with edge–fog–cloud environments, this centralized approach of traditional CDNs could produce latency costs (depending on the characteristics of the network and hardware used for communication) [29]. This latency thus produces delays and awaiting times, which are observed by end-users as large response times [30]. This is crucial in decision-making scenarios [31], where it is expected that the data be available in the shortest possible time (e.g., a physician waiting for data to perform a diagnosis) [32].

Instead of using a centralized cloud storage scheme such as traditional CDNs, in this paper, we propose a hierarchical scheme for the management of data combined with caching techniques to reduce the latency observed when uploading/downloading data to/from the cloud. This hierarchical data management considers the usage of the main memory of the machines as the first option to store and transport data of applications deployed in any of the edge, the fog, or the cloud. This mitigates bottlenecks caused by the allocation and location operations of data when working with distributed environments such as the edge–fog–cloud.

In this paper, we present an efficient scheme for the management and storage of IoT data in edge–fog–cloud environments. This scheme creates continuous dataflows for the delivery and management of IoT data through any combination of the edge, the fog, or the cloud. Dataflows are created by using structures called data containers.

A data container is a self-contained and reusable cache file system service, which includes mechanisms for the management of the input/output data required by the applications considered by an organization/user for processing IoT data transported through the edge–fog–cloud infrastructures. These data management mechanisms were implemented as a cache hierarchical file system that includes three storage levels: in-memory (L1), the host file system (L2), and the cloud (L3). In this sense, data produced by nano/microservices deployed on any of the edge, the fog, or the cloud are cached in local memory as the first option (L1), which reduces the latency costs associated with access to the data when *M* applications are deployed on the same environment. When the local memory space is full, the data containers start to use the file system of the host (managed as a volume in the data container) to temporally store the data. The data are thus sent to the cloud storage in a deferred manner based on caching policies.

The delivery of data through nano/microservices deployed on different environments (any of the edge, the fog, or the cloud) is performed through a content delivery network, which performs the location and allocation of the data required by an application, in automatic and transparent manners.

A prototype of our proposed scheme was implemented and evaluated through a case study, consisting of the management of electrocardiogram sensor data through processing structures deployed on the edge–fog–cloud infrastructures. The experimental results revealed the efficiency of the proposed scheme in comparison with a traditional storage solution implemented using Dropbox.

In summary, the contributions of this work are:

- The design, implementation, and evaluation of an efficient scheme for the continuous delivery and storage of IoT data in edge–fog–cloud environments;
- A hierarchical data management mechanism, included in data containers, to reduce the latency costs associated with the delivery of data in edge–fog–cloud environments.

The rest of the paper is organized as follows. Section 2 presents the related work. Section 3 describes the proposed data scheme for the management of IoT data in edge–fog–cloud environments. Section 5 presents the implementation details of a prototype based on the scheme proposed in this work. Section 6 describes the experimental results from a case study. Conclusions and future research lines are described in Section 7.

## 2. Related Work

In the literature, there are many works focused on data management (including the storage, sharing, and delivery of data) to applications deployed through the edge, the fog, and the cloud. For example, cloud storage solutions as a service, such as Dropbox [33], and content delivery networks (CDN), such as SkyCDS [34] and Amazon CloudFront [35], are storage systems that create replicas of the files that are stored through different storage locations to ensure the availability and distribution of files to the end-users. Nevertheless, the exchange of data through the network creates delays in the delivery of data and contents to the end-users as a consequence of the latency produced in these types of storage solutions.

A static distribution scheme distribution such as RUSH [36] is a family of algorithms that solves the scalability problem by facilitating the distribution of multiple replica objects among thousands of object-based storage devices. Random Slice (RS) [37] is a data distribution strategy that incorporates lessons learned from table-based and pseudo-random hashing strategies to be fair and efficient in homogeneous and heterogeneous environments to adapt and change storage containers. CRUSH [38] is a pseudo-random data distribution algorithm that efficiently and robustly distributes replicas across heterogeneous and structured clusters. RS-Pooling [39] is an adaptive random data distribution strategy for fault-tolerant, large-scale storage systems. Moreover, a scheme distribution dynamic such as AREN [40] is a replication scheme for cloud storage based on bandwidth and a collaborative cache strategy to provide a number of replicas of the popular content. DPRS [41] is a data replication strategy that places popular files in appropriate clusters/sites to adapt the caching of files based on the user interests considering the number of requests, and the distribution of requests over time. CDRM [42] is a scheme for cloud storage, which builds a model to capture the relationship between availability and replication number, taking into account the capacity (CPU power, memory capacity, network bandwidth, etc.) and blocking probability of each data node.

In this sense, different distribution schemes have been developed and deployed as a middleware between the end-users applications and the cloud storage systems (e.g., Dropbox and SkyCDS). For example, GlusterFS [43] is a distributed file system that provides shared and replicated storage across multiple storage locations. It implements a shared storage system that reduces the latency to exchange data between different storage locations. IRIS [44] is a unified and integrated storage access system implemented as a middleware that unifies the data model and the underlying storage framework. These middlewares abstract the access to the data by end-users and applications. Nevertheless, these tools lack of mechanisms for the efficient management of data based on caching techniques and in-memory data management.

Alluxio [45] provides hierarchical storage that performs the data allocation and location tasks through distributed environments. It implements a caching mechanism that automatically moves the data close to the applications in HDFS (Hadoop Distributed File System) systems. Hermes [46] is a heterogeneous I/O buffering system that manages and monitors data based on in-memory storage mechanisms. Similar, RAMCloud Storage System (RCSS) [47] is an HDFS-based in-memory storage system that improves the performance of input/output systems in HDFS [48] systems.

Table 1 presents a summary of the different distribution schemes and storage resource management for the transportation of data through any of the edge, the fog, or the cloud. As can be observed, most of the works are focused on the management of data in the fog and the cloud, where the resources available have higher computational and storage capabilities. In turn, the work proposed in this paper considers the transporting of data through any environment. Data containers proposed in this paper have the characteristic of portability, which means that a container can be moved and deployed through different platforms and infrastructures for the management of data.

Data storage management refers to how the data are stored in the storage systems. Thus, the data can be stored as files, blocks, or objects [49]. Solutions such as RS and IRIS store the data in the form of files by following hierarchical management of the files, where files are organized in a tree of nested folders. Nevertheless, when working with distributed environments, the scaling of systems based on files is complex as is required a central component for the management of the hierarchy of files and directories. In turn, object-oriented storage systems are easy to scale, as the metadata and an identifier of the data are stored with the data in a single self-contained object. This reduces the complexity to locate and allocate data through a distributed storage system. The data containers proposed in this paper produce objects instead of files, similar to solutions such as RUSH, RS-Pooling, AREN, and Alluxio.

In the literature, there are few solutions with hierarchical storage management of data, including the memory, the filesystem, and the cloud, similar to that implemented in the data containers proposed in this paper. These tools are Alluxio, IRIS, and Hermes. To manage the caching through this hierarchy, these solutions apply two policies: last frequently used (LFU) [50] and last recently used (LRU) [51]. In LFU, the most accessed data are moved to the top of the hierarchy, whereas in LRU, the newest data are moved.

## 3. Design Principles of an Efficient Scheme for the Management and Storage of IoT Data

In this section, we described the proposed scheme for creating continuous dataflows to efficiently deliver and store IoT data in edge–fog–cloud environments. These dataflows are built by using entities called *data containers*, which are attached with nano/microservices developed for the acquisition, processing, and production of data in edge–fog–cloud environments. Sets of data containers are chained to create continuous dataflows through edge–fog–cloud infrastructures.

This section also presents a hierarchical data management included in virtual containers to reduce the latency costs associated with the management of data in edge–fog–cloud environments.

### 3.1. Data Containers for the Efficient Management and Delivery of Data in Edge–Fog–Cloud Environments

The basic data management unit of the scheme proposed in this paper are software entities called data containers. Data containers enable organizations to establish controls over the exchange of IoT data produced/required by applications implemented in the form of nano/microservices for the management/processing of data in any of the edge, the fog, or the cloud. The main goals of data containers are:

- To efficiently and transparently manage the data produced/managed by applications deployed on edge–fog–cloud environments;
- To create continuous dataflow in the edge–fog–cloud by the interconnection of data containers distributed through any of the edge, the fog, or the cloud;
- To reduce the latency costs associated with the storage of data in the cloud observed in traditional content delivery networks (CDNs).

To achieve these goals, in this scheme, the data containers are built as self-contained software pieces that include mechanisms for the efficient management of data produced/required by edge–fog–cloud applications. In this scheme, a data container is implemented as a virtual container with storage and memory limitations. A data container thus includes storage spaces in the memory and file systems (e.g., hard disks) for temporally allocating data to reduce the costs associated with the transference of data directly to the cloud.

**Figure 1:**
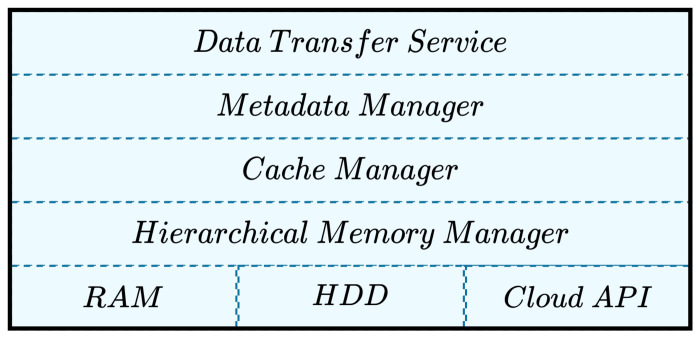
Stack representation of a data container for the efficient management of data.

Figure 1 depicts an architecture stack of a data container (DC) and its components. The first layer includes a *data transference service manager* that is in charge of managing the data arriving/departing (input/output data) to/from a data container. This layer also includes an *access control layer*, which verifies that the tokens and credentials for ensuring that only authorized users/applications have access to the input/output data managed by the data container. Data containers also include a metadata manager, which is in charge of establishing controls over the data allocated and located in a data container. A *cache manager* implements data caching policies to add/delete data from each component considered in the *hierarchical memory manager*, which is the last layer considered in the stack of a data container.

The *hierarchical memory manager* is in charge of managing the storage of data produced/required by an application. This manager implements a hierarchical file system divided into three levels:

- *Level 0 (L0) or local memory (RAM)*: in this level, the local memory attached to the data container is used to temporally store data, before being written to disk (level 1). This level is more convenient when multiple applications deployed in a single environment are exchanging data. In this sense, application 1 must deliver the memory address to application 2 so it can retrieve data. These operations are performed by the data container, not by the applications. For example, in a node in the fog, a data preprocessing application delivering contents to a deep learning application [52], the delivery and retrieval of data are performed using the memory by a data container that performs these operations as a middleware.
- *Level 1 (L1) or local storage (host filesystem)*: in this level, data are stored in the file system of the data container host (i.e., hard disk). At this level, data are temporally stored by using a deferred data migration scheme.
- *Level 2 (L2) or cloud storage*: in this level, the data are stored in the cloud by using a content delivery network (CDN), which is based on a pub/sub scheme and implements authentication and load-balancing mechanisms. Thus, the CDN is in charge of automatically distributing the contents required by applications deployed on any of the edge, the fog, or the cloud.

The *cache manager* is in charge of caching data through this hierarchical file system. To this end, two caching policies are available in the data containers to add/remove data to/from each level of the hierarchical storage: last frequently used (LFU) and last recently used (LRU). In LFU policy, the less accessed data are deleted from the cache (L0 or L1) and sent to the next storage level (L0 → L1 | L1 → L2). In turn, in LRU policy, the most recently used data are stored in the top levels of the hierarchical filesystem (L0 or L1), whereas the oldest produced data are moved to the lower levels (L2).

### 3.2. Creating Storage Systems Based on Pools of Data Containers

At this point, we have presented the design of data containers for the efficient management of I/O data required/produced by applications deployed in any of the edge, the fog, or the cloud. In real scenarios, applications distributed through the edge–fog–cloud require exchanging data to process them and produce insights and information useful in decision-making scenarios. In this scheme, data containers, deployed on edge–fog–cloud infrastructures, are organized into a data pool that transparently manages the I/O access to data by creating a temporal storage service based on a distributed caching system. In this service, data are transparently exchanged among the infrastructures by using a CDN.

**Figure 2:**
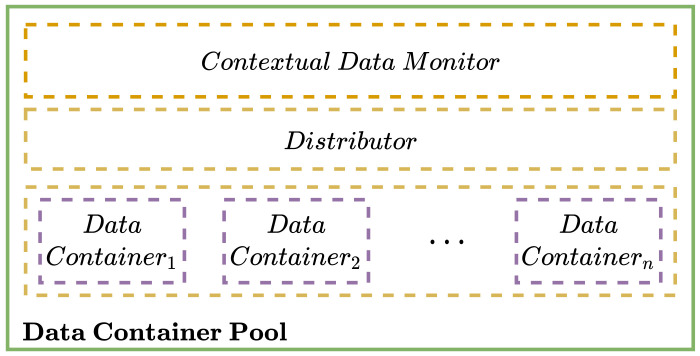
Stack representation of a temporal storage service created by using a data container pool.

Figure 2 shows the stack representation of a pool of temporal storage services created by using a pool of data containers. This is composed of a contextual data monitor, a distributor, and the data containers.

As it can be observed, data containers are grouped in a pool, where data allocation/location operations are managed by a data distributor. A data contextual monitor performs the continuous monitoring of data containers as well as the applications attached to these containers. This monitor collects performance metrics such as memory utilization, size of the outputs produced by an application, size of the inputs required by an application, file system utilization, and the number of accesses to a file. These metrics are delivered to the data distributor and data container pool to manage the caching of files in the data container file system.

The data distributor is in charge of performing the allocation and location of data in the data container pool. This component follows a ball-into-bins metaphor to perform the distribution (allocation) of data through the data containers available in the pool. This distributor includes load-balancing mechanisms to produce a fair distribution of data between data containers launched in a pool. The load-balancing algorithms available are:

- *Round Robin*: cyclically distributes the contents through the available data containers, which have the same probability of being elected. The data container, where the data will be stored, is elected as follows:
  (1) dc=(imodN)|i∈N,
  where dc is the data container elected, *i* is a numeric identifier of the file to be allocated, and *N* is the total number of data containers available.
- *Random*: randomly, an available data container is elected to store the data. In this algorithm, each data container has the same opportunity to be elected.
- *SortingUF* [53]: the utilization factor (UF) of each data container is calculated based on the used storage and memory. The data containers are sorted based on their UF, where the data container with the lowest UF is the one elected to store the data.
- *Two Choices* [54,55]: in this algorithm, two data containers are randomly elected, and the data are stored in the data container with the lowest storage utilization.

Algorithm 1 presents the process of allocating and locating data in a pool of data containers. This Algorithm receives as input the data (*D*) to be allocated in the *n* available data containers of the data container pool (*DP*), the operation (*op*) of either allocation or location, as well as the load-balancing technique to distribute data (*LBalgorithm*). The output of the Algorithm 1 is a set of maps in the form of < *d,dc,dataHash* >, where *d* is the data to allocate, *dc* is a data container available in the *DP*, and *dataHash* is the digital signature of the data, which is unique for each *dc*.
**Algorithm 1** Allocation/location of data in a pool of data containers.**Require:** data (*D*), netadata list (*ML*), operation type (*op*), load balancer algorithm    (*LBalgorithm*), data container pool (*DP*)**Ensure:** maps of data allocation/location (*mapsAL*)  1:*dataHash* = *""*  2:*exist* ← *NULL*  3:*mapsAL* ← {}  4:*index* = 0  5:**for all***d* ∈ *D* **do**  6:    *dataHash* ← *calculateHash*(*d*)
  7:    *exist* ← *data.exist*(*dataHash,mL*)
  8:    **if** *op* == *"allocation"* **then**
  9:        **if** *exist* == *FALSE* **then**
10:            *mapsAL*[*index*] ← *LB*(*LBalgorithm,dataHash,DP*)
11:            *data.recordD*(*ML,mapsAL*)12:            *data.allocation*(*d,mapsAL*)
13:            *index*++14:        **else**15:            **This file already exists**16:        **end if**17:    **end if**18:    **if** *op* == *"location"* **then**19:        **if** *exist* == *TRUE* **then**20:            *mapsAL*[*index*] ← *data.location*(*dataHash,ML*)21:            *index*++22:        **else**23:            **This file hasn’t been located**24:        **end if**25.    **end if**26:**end for**27:**return***mapsAL*

## 4. Continuous Dataflows for the Delivery of Data through Data Containers in the Edge–Fog–Cloud

In a differed manner, the data stored in a data container pool are sent to the cloud by using a CDN based on catalog abstractions, which are basically virtual storage spaces in multi-cloud environments. Moreover, this CDN interconnects data containers deployed in any of the edge, the fog, or the cloud, enabling the exchange of data and creating continuous edge–fog–cloud dataflows.

**Figure 3:**
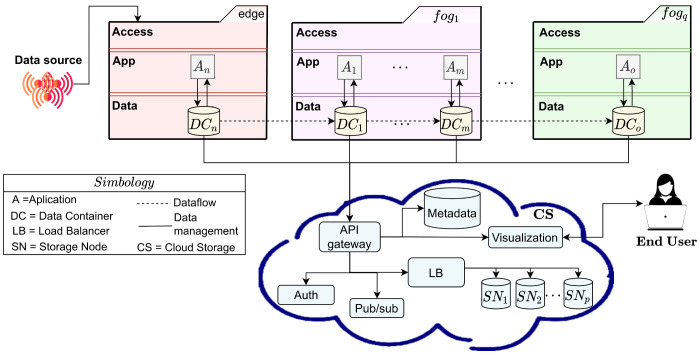
Efficient data delivery scheme for the edge–fog–cloud.

Figure 3 shows the conceptual representation of a dataflow created with data containers in an edge–fog–cloud infrastructure. As it can be observed in Figure 3, data containers are in charge of the management of input/output data required/produced by applications deployed on any of the edge, the fog, or the cloud (see $A_{n}\in \mathrm{edge}$, $A_{1,\;\cdots ,\;m}\in fog_{1}$, and $A_{o}\in fog_{q}$ in Figure 3).

The CDN is based on a pub/sub model, where users and applications can subscribe/publish catalogs storing data (e.g., ECG signals). The metadata of the data managed by catalogs are registered in a database, and an authentication module is in charge of managing the access control to the catalogs. The CDN also implements a load-balancing mechanism to distribute the incoming data through the available storage nodes (see $SN_{1}$, $SN_{2}$, and $SN_{p}$ in Figure 3). End-users can consume the data stored in the catalogs through a visualization client, which enables them to perform subscription operations that trigger a synchronizer, which automatically downloads the data to their computer.

## 5. Prototype

This section describes the implementation of a prototype for creating edge–fog–cloud dataflows based on the proposed scheme. Data containers are managed as Docker virtual containers and implemented in Java programming language. In this scheme, the applications producing/consuming data to/from data containers are managed as nano/microservices encapsulated into virtual containers. The communication of the data containers and applications is performed by using inter-process communication (IPC) through shared memory. The exchange of messages between data containers is performed through a remote procedure call (GRPC) [56].

The caching mechanism included in data containers is implemented in Java programming language. The context data monitor, included in the data container pool, is implemented by using cAdvisor, which is a tool to perform the monitoring of Docker containers [57]. The CDN integrated in this scheme is implemented as a microservice (encapsulated into virtual containers) and coded by using PHP7. This CDN is thus composed of five microservices: Authentication, pub/sub, load-balancing, metadata, and visualization, as well as services for the management of the storage nodes and an API gateway to manage the incoming requests to the CDN. The databases of the CDN are implemented by using PostgreSQL.

## 6. Experimental Evaluation

In this section, we present the experimental evaluation conducted in the form of a case study to evaluate a prototype based on the proposed scheme. This case study considers applications in the edge–fog–cloud for the processing and management of electrocardiogram (ECG) data by using data analytic microservices.

**Figure 4:**
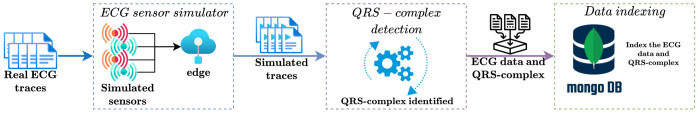
Conceptual representation of the structure used to perform the case study of the experimental evaluation.

Figure 4 presents the design of this case study, which considers the following microservices for the management of processing and analysis of ECG data:

- *ECG sensor simulator*: this application receives as input a set of five real ECG traces. At the start, a trace is randomly elected to periodically produce ECG data packages by reading the measurements contained in the selected trace and adding a timestamp as well as an identifier. These ECG data packages are written to a new file, which is the output of this application. The application was developed in Python and can be tuned to modify the total number of sensors to simulate, the number of packages to write in the output trace, and the time in seconds to wait between the generation of each package. This application can be deployed on any computer on the edge to simulate a HUB receiving data from real ECG sensors.
- *QRS-complex detection*: this application, developed in Python, processes the ECG traces produced with the simulator to identify QRS-complex in the data [58].
- *Data indexing*: this application, developed in Python, receives the ECG data and the QRS complex generated with the previous applications. The received data are indexed in a MongoDB database.

To conduct this experimental evaluation, we implemented the processing structure depicted in Figure 4 to manage the exchange of data with the scheme proposed in this paper and a traditional storage service implemented by using Dropbox. We divide this evaluation into two phases. In the first one, a controlled evaluation was conducted by transferring ECG sensor traces between two nodes by tuning the parallelism degree, the size of the traces, and the number of traces to exchange. In the second one, the performance of the proposed scheme was evaluated when managing ECG sensor data through the structure depicted in Figure 4.

### 6.1. Environment of Experimentation

Table 2 shows the main hardware characteristics of the infrastructure used to conduct each experiment considered in this experimental evaluation. In experiments 1, 2, and 3, we used two computers to evaluate and tune the performance of data containers by exchanging data between two applications (sensor simulator and QRS detector) deployed in a fog node labeled as Compute 1. A machine labeled as Compute 2 was used as a cloud storage node to store the data produced by the sensor simulator. To conduct these experiments, the sensor simulator was configured to generate trace files of 1 and 10 MB.

In the fourth experiment, three machines were used to emulate the edge–fog–cloud scenario depicted in Figure 4, considering applications deployed in edge (labeled as Compute 3 in Table 2) and fog (labeled as Compute 4 in Table 2) nodes. In the edge node the sensor simulator application was deployed, whereas in the fog the QRS detector and data indexing applications were deployed. A cloud storage node labeled as Compute 5 was used to store the data produced by these applications considered in each stage. In this experiment, the sensor simulator was configured to produce 10,000 packages (measurements).

### 6.2. Tuning the Parameters of Data Containers in a Controlled Evaluation

In this phase, we evaluated the performance of different configurations in the data containers proposed in this scheme. This evaluation was conducted by exchanging data between two stages (the sensor simulator uploading data and the QRS-complex detector downloading the data). Both applications were deployed in a fog node (Compute 1). The goal of this phase is to evaluate the behavior of the solutions evaluated by testing different configurations varying the degree of concurrency, the size of the traces to exchange, and the number of traces. In addition, this evaluation includes a configuration using a traditional storage service implemented by using Dropbox. The following configurations were evaluated:

- **Data containers—Configuration 1**: a solution managing data with the data containers configured with a cache of 40 pages and 2 GB of available memory.
- **Data containers—Configuration 2**: a solution managing data with the data containers configured a cache of 100 pages and 24 GB of available memory.
- **Traditional storage service**: a solution implemented by using a Dropbox client in Python to exchange data between two nodes.

**Figure 5:**
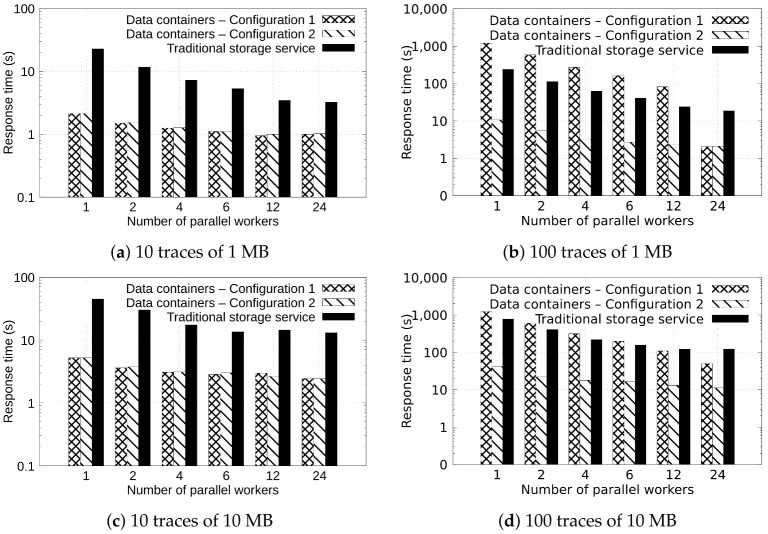
Response time observed when transferring 10 and 100 traces of 1 and 10 MB by using a varying number of parallel workers.

Figure 5a–d show, in the vertical axis, the response time of configurations processing the exchange of 10 and 100 ECG files of 1 and 10 MB size each. Different configurations were defined by varying the degree of parallelism (horizontal axis) defined by the number of *workers* (number of microservices processing the ECG files). This means that a bigger number of parallel workers represents more clients transferring files through the processing structure. The ECG trace files are distributed to each worker in a parallel manner. As expected, the bigger the parallelism degree, the greater the reduction in the response times. For example, in Figure 5d, we can observe that the exchange of 10 ECG files of 10 MB by using the data containers solutions with one worker spent 5.19 s, whereas with four workers, it spent 3.01 s. This represents a performance gain of 67.23%. Similar behavior is observed when using the different configurations of a traditional storage service and when increasing the number of ECG files to exchange data through the structure. In Figure 5d, it can be observed that 100 ECG files of 10 MB were exchanged in 41.40 s by using data containers solution with one worker. Meanwhile, with four workers, the response time is reduced to 17.88 s, representing a performance gain of 56.80%.

In Figure 5a–d, we can also observe that when the size of the memory and number of cache pages available increases, the response time of the data containers is reduced as all data is managed at the first level of the data container file system. For example, when transferring 100 ECG files with Configuration 1 of data containers with one worker, the response time observed was 1227.63 s, whereas when increasing the size of the memory and cache available, the response time was reduced to 41.40 s. This is a percentage of the performance gain of 96.62%.

In addition, in Figure 5a–d, it can be observed that the Configuration 2 of the data containers yields a lower response time than the traditional storage service solution. For example, the traditional storage service solution spent 777.52 s to exchange 100 ECG files of 10 MB with one worker (see Figure 5d), whereas the data container solution spent 41.40 s. This is an acceleration of 18.77X and a percentage of performance gain of 94.67%.

### 6.3. Analyzing the Cache Usage in Data Containers

**Figure 6:**
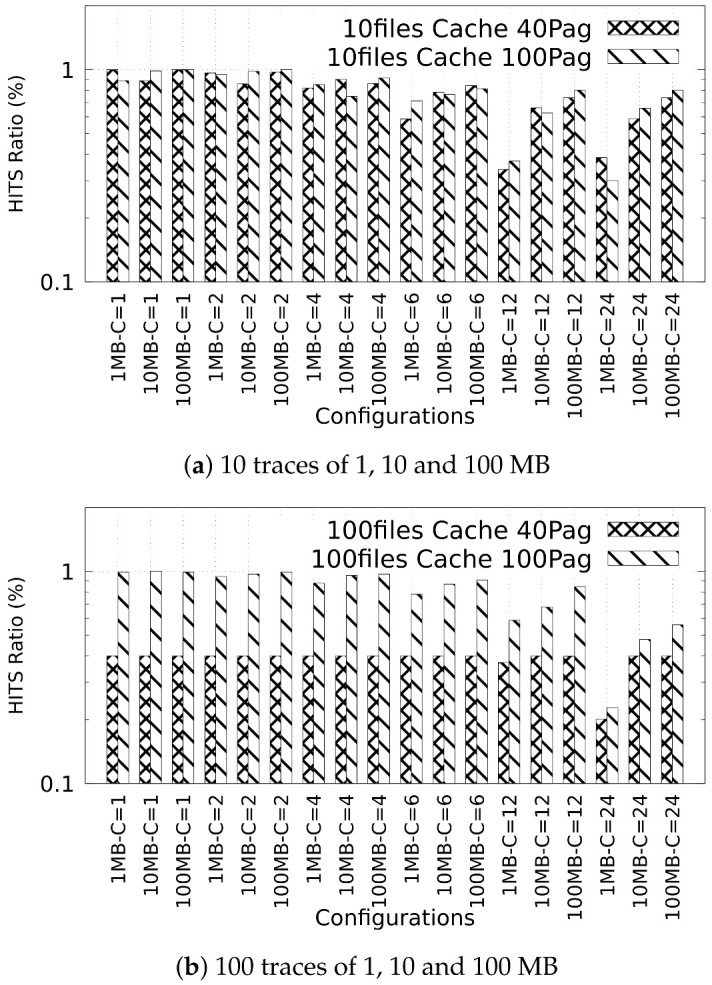
Percentage of hits ratio in the cache observed when exchanging data between two fog nodes.

In this experiment, we analyzed the cache usage in data containers to perform the operations of data allocating and locating when exchanging 10 and 100 ECG traces of 1, 10, and 100 MB in size through two stages in a fog node. Figure 6 depict the cache hits ratio (in a range of 0 to 1) of data requests that have been successfully served by the cache. In this context, while the value is close to one, it means a higher success rate in the data requests performed by the stages exchanging data.

Figure 6a,b show, in the vertical axis, the hit ratio to 10 and 100 allocated and located files of 1, 10 and 100 MB when two different cache sizes (40 and 100 pages) are used, as well as by using different configurations of concurrency (*C*) (see horizontal axis in Figure 6a,b).

A number close to or equal to 1 in the hits ratio means a higher use of the available cache pages; thus, more memory is being used to transfer the data and the contents are not being written in the bottom hierarchy of the file system (L1 and L2). For example, when allocating 100 files of 100 MB with Configuration 1 of data containers with a concurrency equal to 1, the hit ratio was 0.4, whereas when increasing the cache pages available (Configuration 2), the hit ratio increases to 0.99. This means that the usage of the cache is increased by 59%. This reduces the latency observed when exchanging data between stages. Moreover, we can observe that when the concurrency increases, the hit ratio decreases. For example, if the concurrency increases from 1 to 24, then the hit ratio decreases to 0.5 for the last configuration, representing that the cache usage is increased to 43%.

### 6.4. Evaluating the Upload and Download of Data Operations

In this experiment, we evaluate the service time to perform the operations of data uploading and downloading. Again, to perform this experiment, we use two stages (ECG sensor simulator and QRS-complex detector deployed in a fog node labeled as Compute 1). The uploading of the data was evaluated using the ECG sensor simulator stage, whereas the downloading of the data was evaluated by using the QRS-complex detection stage. To perform this evaluation, we used the traditional storage service and Configuration 2 of data containers, which were the solutions that yield the best performance in previous experiments.

**Figure 7:**
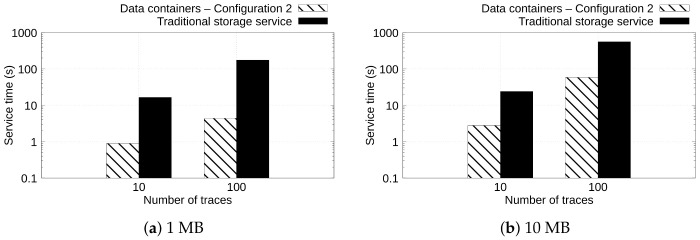
Service time observed when uploading 10 and 100 traces of 1 and 10 MB.

**Figure 8:**
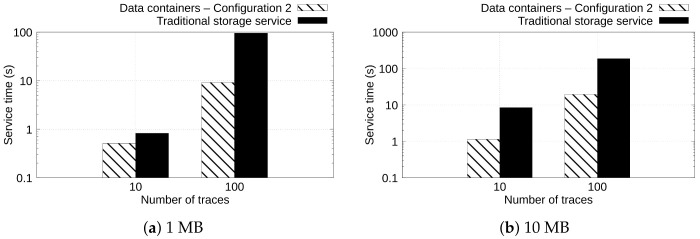
Service time observed when downloading 10 and 100 traces of 1 and 10 MB.

Figure 7 and Figure 8 depict the service time observed when uploading and downloading 10 and 100 ECG files of 1 and 10 MB with Configuration 2 of data containers and the traditional storage service. The goal of these experiments is to show the behavior when uploading and downloading a different number of files.

Figure 7 shows, in the vertical axis, the service time, in seconds, spent by configurations to upload 10 and 100 ECG files (horizontal axis) of 1 and 10 MB (see Figure 7a,b, respectively). Again, we can observe that when the data containers are configured with a large amount of memory and a higher number of cache pages, the service time to upload the data is reduced in comparison with traditional cloud storage such as Dropbox. For example, in Figure 7b, can be observed that *Data containers—Configuration 2* upload 100 ECG files of 10 MB in 24.11 s, whereas the traditional storage service uploads the same content in 561.09 s. This means a 95.70% reduction in the service time of the data containers in comparison with the traditional storage service.

Figure 8 shows in the vertical axis the service time observed when uploading 10 and 100 traces (horizontal axis) of 1 and 10 MB (see Figure 8a and Figure 8b, respectively). Similar behavior was observed in Figure 8 when downloading contents. Again, *Data containers—Configuration 2* yields a lower service time than the traditional storage service solution. For example, in Figure 8b, it can be observed that *Data containers—Configuration 2* downloaded 100 ECG files of 10 MB in 8.44 s, whereas the traditional storage service downloads the same workload in 185.40 s. This represents that the data containers configuration yields a performance gain of 95.44% in comparison with the traditional storage service.

### 6.5. Exchanging Data through Multiple Stages

In this experiment, we evaluate the performance of the studied solutions when managing data through a structure considering three stages: ECG sensor simulator, QRS-complex detection, and data indexing. The ECG sensor simulator was deployed on an edge node (labeled as Compute 3 in Table 2), whereas the QRS-complex detection and data indexing were deployed on a fog node (labeled as Compute 4 in Table 2). In this experiment, a cloud storage location (labeled as Compute 5 in Table 2) was configured for the storage of the data.

**Figure 9:**
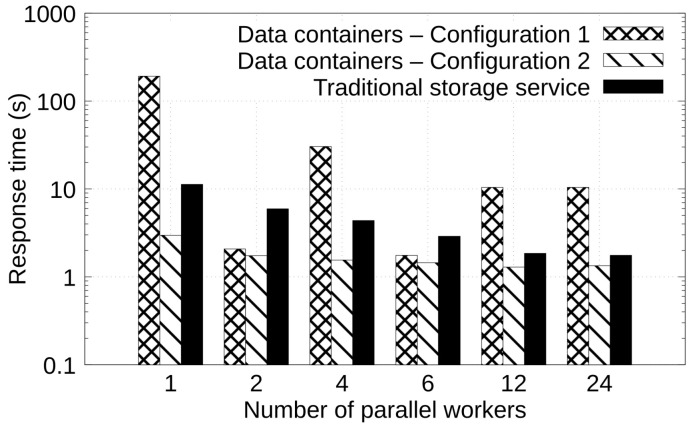
Response time when managing ECG data through a structure considering three stages: ECG sensor simulator, QRS-complex detection, and data indexing.

Figure 9 shows in the vertical axis the response time, in seconds, observed for the management of 10 ECG traces when varying number of workers (horizontal axis). Again, we can observe that as more workers are added in the processing stages (ECG sensor simulator, QRS-complex detection, and data indexing), the response time is reduced. In addition, it can be observed that in a configuration with a higher number of pages in cache (Data containers—Configuration 2), the response time is also reduced. For example, with 12 workers, Configuration 1 and the traditional storage service yields a response time of 10.43 and 1.85 s, respectively, whereas Configuration 2 performs the same operations in 1.29 s. This represents a reduction in the response time of 87.57% and 30.15% in comparison with Configuration 1 and the traditional storage service solutions, respectively.

## 7. Conclusions

In this paper, we presented the design, development, and evaluation of an efficient scheme for the management and storage of IoT data in edge–fog–cloud environments. This scheme includes entities called data containers, which manage the input/output data required/produced by applications deployed on edge–fog–cloud infrastructures. These data containers implement a hierarchical cache file system including three storage levels: in-memory, filesystem, and the cloud. Data containers are organized in the form of data pools to create temporal storage services to distribute contents between applications distributed in any combination of the edge, fog, or cloud computing resources.

The experimental evaluation conducted in the form of a case study for the management of ECG data revealed, in a direct comparison with a traditional storage service, the efficiency of the proposed scheme to manage data in edge–fog–cloud scenarios.

We are considering as future work the conduction of large-scale study cases of the management of medical and satellite contents by integrating a serverless scheme for the creation of storage systems for serverless applications (e.g., function as a service [59]) deployed in endpoints on any of the edge, the fog, or the cloud. Moreover, we are working on the integration of data preparation schemes on the client side for the management of crucial non-functional requirements in the management of sensitive data (e.g., security, reliability, and traceability).

## Figures and Tables

**Table 1:** Summary of storage tools for the data management and distribution schemes.

Work	Scope	Infraestructure			Portability	Data Storage Management			Hierarchical Storage			Caching Policy	
		Edge	Fog	Cloud		Files	Blocks	Objects	Memory	FS	CS	LRU	LFU
RUSH (2004) [36]	DS	-	✓	✓	-	-	-	✓	-	✓	-	-	-
RS (2014) [37]	DS	-	✓	✓	✓	✓	-	-	-	✓	-	-	-
CRUSH (2006) [38]	DS	-	✓	✓	-	-	-	✓	-	✓	-	-	-
RS-Pooling (2016) [39]	DS	-	✓	✓	-	-	-	✓	-	✓	✓	-	-
AREN (2012) [40]	DS	✓	-	✓	-	✓	-	✓	-	✓	✓	-	-
DPRS (2017) [41]	DS	-	✓	-	-	✓	✓	-	-	✓	-	✓	-
CDRM (2010) [42]	DS	-	-	✓	-	✓	-	-	-	✓	✓	-	-
GlusterFS Container (2016) [43]	SRM	-	✓	✓	✓	✓	-	-	-	✓	-	-	-
Alluxio (2018) [45]	SRM	-	-	✓	-	✓	✓	✓	✓	✓	✓	✓	-
IRIS (2018) [44]	SRM	-	✓	✓	-	✓	-	✓	-	✓	✓	✓	✓
Hermes (2018) [46]	SRM	-	✓	✓	-	✓	-	-	✓	✓	✓	✓	✓
Proposed data scheme	SRM & DS	✓	✓	✓	✓	-	-	✓	✓	✓	✓	✓	✓

DS = Distribution scheme. CS = Cloud Storage. SRM = Storage resources management. FS = Filesystem.

**Table 2:** Characteristics of the infrastructure used to conduct the experimental evaluation.

Experiment	Label	Role	Hardware Characteristics				Software Deployed	
			# Cores	CPU	RAM (GB)	Storage	Application	Role
1, 2, and 3	Compute 1	Fog node	24	Intel Xeon CPU E5-2650	256	3.7 TB	Sensor simulator	Producer
							QRS detector	Consumer
	Compute 2	Cloud node	24		128	2.7 TB	Cloud storage	Storage
4	Compute 3	Edge node	12		64	17.5 TB	Sensor simulator	Producer
	Compute 4	Fog node	24		256	3.7 TB	QRS detector	Consumer and Producer
							Indexing	Consumer and Producer
	Compute 5	Cloud node	24		128	2.7 TB	Cloud storage	Storage

## Data Availability

Not applicable.
